# Data on differentially expressed microRNAs in the liver between nonalcoholic fatty liver disease and normal Wistar rat using Solexa sequencing

**DOI:** 10.1016/j.dib.2016.05.041

**Published:** 2016-05-24

**Authors:** Deqing Zhang, Yuqian Wang, Zhibin Ji, Zhonghua Wang

**Affiliations:** a Shandong Key Laboratory of Animal Biotechnology and Disease Control and Prevention, College of Animal Science and Technology, Shandong Agricultural University, Taian 271018, PR China; b Laboratory Department, Taian Central Hospital, Taian 271000, PR China

**Keywords:** MicroRNAs, Nonalcoholic fatty liver disease, Rat, Solexa sequencing

## Abstract

The dataset includes data from the Solexa sequencing reported in our paper: “Identification and differential expression of microRNAs associated with fat deposition in the liver of Wistar rats with nonalcoholic fatty liver disease” [1]. The data collected include small RNAs and microRNAs in liver tissue from high glucose-induced NAFLD Wistar rats, using normal Wistar rats as their negative controls. 6 small RNA libraries were constructed and the expression profiles were compared between the two groups. Here we describe in detail how the data, deposited in the Gene Expression Omnibus (GEO) with the accession number GEO: GSE68411, was generated including the basic analysis.

**Specifications Table**

**Table:** 

Subject area	Biology
More specific subject area	Animal nutrition and metabolism
Type of data	Figure and Table
How data was acquired	Solexa sequencing of Illumina HiSeq 2000 Genome Analyzer and Quantitative PCR analysis was performed
Data format	Filtered and analyzed
Experimental factors	3 NAFLD rat liver tissue samples and 3 normal rat liver tissue samples were used for RNA sequencing.
Experimental features	Rat model of NAFLD were successfully established and three biological repetitions were performed to reduce the variance caused by individual differences. The livers of NAFLD group, chosen from among the samples with higher levels of hepatic triglycerides, and those from normal group, chosen randomly, were subjected to Solexa sequencing.
Sample source location	Experimental Animal Center of Shandong University, Jinan, China.
Data accessibility	Data are provided in the paper

**Value of the data**

• The data may provide these miRNAs differentially expressed in the liver as the potential biomarkers of NAFLD.

• The data lays a solid foundation for the function study of novel miRNAs involved in the pathophysiology of NAFLD for further.
• The data of GO annotation and KEGG pathway analysis indicates that some miRNAs play important roles in the process of liver fat deposition.
• The data may be useful as comparison with human liver miRNAs studies of NAFLD.

## 1 Data

The dataset described consists of cleaning reads data and the data of standard bioinformaitcs analysis. The cleaning reads data contains clean reads and the length distribution of small RNA after removing adaptors, low quality reads as well as contaminants (Supplementary Table 1, 2). The data of standard bioinformaitcs analysis mainly includes known miRNAs, novel miRNAs and its secondary structure by Mireap (Supplementary Table 3), differential expression analysis of known miRNAs (Supplementary Table 4), target genes prediction for novel miRNAs (Supplementary Table 5), KEGG pathway annotations and GO annotations for the target genes (Supplementary Table 6,7).

## 2 Experimental design, materials and methods

### 2.1 Experimental design

A rat model of NAFLD was established and the liver tissues of NAFLD Wistar rats and normal Wistar rats were subjected to Solexa sequencing. Standard bioinformatics analysis was performed to annotate the clean reads into different categories. 6 Small RNA libraries were constructed, and the expression profiles of miRNAS were compared between the both groups.

### 2.2 Materials and methods

#### 2.2.1 Establishment of a NAFLD Wistar rat model

Twenty-four male Wistar rats, aged 28 weeks old and weighing 445–560 g were randomly divided into two groups: a high- glucose group (HG) and a normal group (NG). All rats were kept on a 12 h light/dark cycle and fed ad libitum food and water during the whole study. HG Wistar rats were first fasted for 48 h, and then fed ad libitum with a diet containing starch (80%), casein (16%), and mineral and vitamin mix (4%), whereas NG rats were fed standard diet containing 69% carbohydrate (including 35% starch), 18% protein, 4% fiber, 3% lipids, and 5% minerals and vitamins. At the end of the 7th day, all the rats were sacrificed by removal of the liver under deep ether anesthesia. The levels of hepatic TG were routinely determined with TG kits. The liver was processed into paraffin sections using standard methods and sections were dyed with hematoxylin and eosin staining and observed under an optical microscope.

#### 2.2.2 Solexa sequencing

The liver tissues of HG-induced NAFLD Wistar rats chosen from among the samples with higher levels of hepatic triglycerides, and those from normal Wistar rats chosen randomly, were frozen immediately in liquid nitrogen after dissection. Total RNAs were extracted using TRIzol reagent and the quality of the total RNA was examined using gel electrophoresis (28S:18S>1.5) and a bioanalyzer. Total RNAs were stored at –80 °C.

**Fig. 1:**
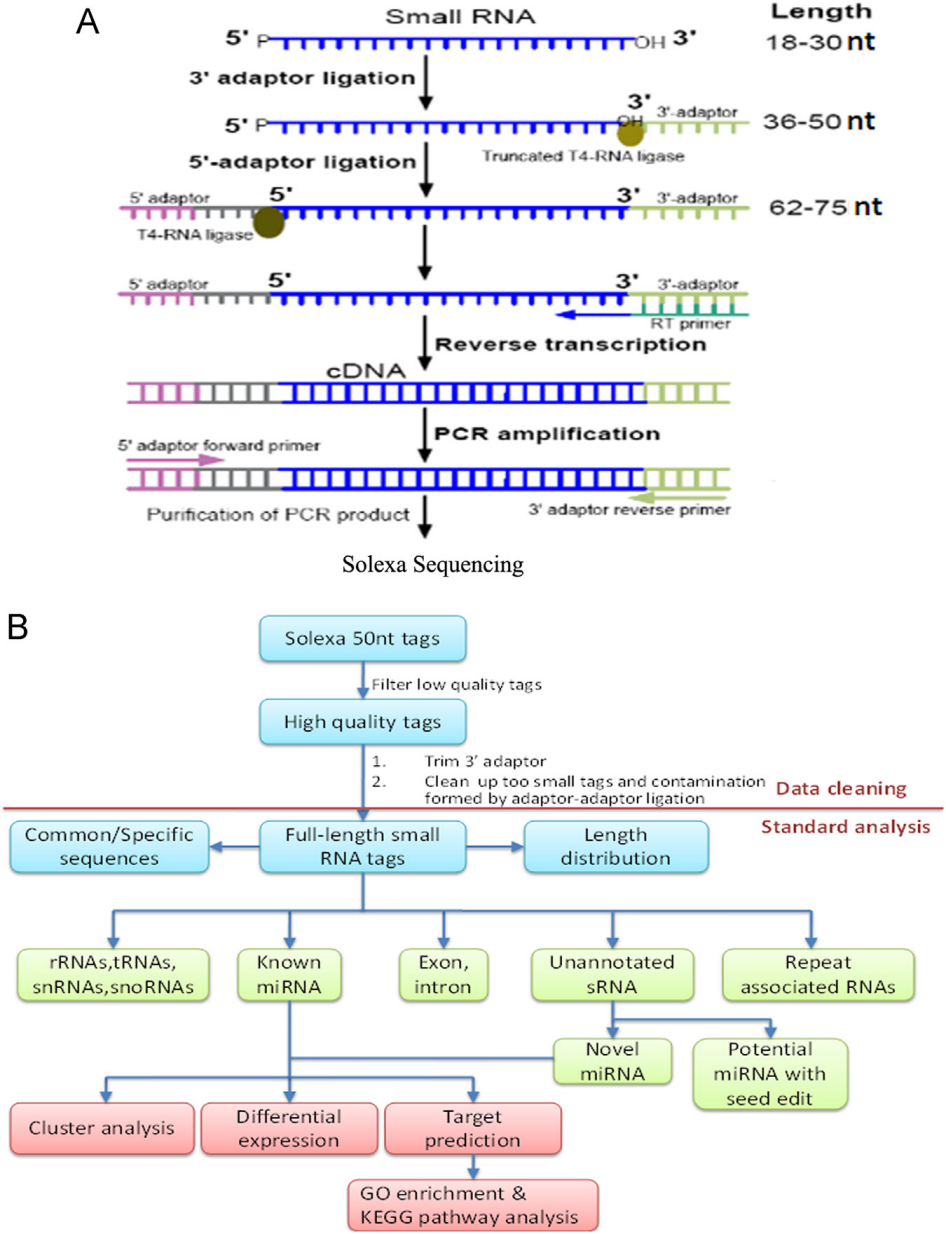
The overall process of Solexa sequencing. A experimental process of small RNA sequencing. The small RNAs were converted to cDNA by RT-PCR. PCR products were purified and prepared for Solexa sequencing. B Solexa sequencing data analysis**.** The 50 nt reads from Solexa sequencing were first subjected to data cleaning. Standard bioinformatics analysis was used to annotate the clean reads into different categories; those that could not be annotated into any category were used to predict novel miRNAs. (TIF).

The total RNA of each sample was processed using a Small RNA Sample Prep Kit in accordance with the kit instructions. Briefly, the 18–30 nt fragments of the total RNA were excised and purified using polyacrylamide gel electrophoresis. Next, a pair of Solexa adaptors was ligated to the 5׳ and 3׳ ends of the small RNA, respectively, using T4 RNA ligase. The adapted small RNAs were converted to cDNA by reverse transcription and the cDNAs were amplified by PCR. The PCR products were purified through 4% agarose gels and were prepared for Solexa sequencing. Finally, the image files generated by the Solexa sequencer were processed to obtain quality digital data (Fig. 1A). Fig. 1B shows the overall flow of the bioinformatics analysis.

#### 2.2.3 Bioinformatics analysis

All raw reads obtained were processed according to the rules of Sunkar et al. [2] as follows: i) removal of low quality reads; ii) removal of reads with 5׳ primer contaminants; iii) removal of reads without 3׳ primers; iv) removal of reads without the insert tag; v) removal of reads with poly A; vi) removal of reads shorter than 18 nt; and vii) summarization of the length distribution of the clean reads. The clean reads were mapped to the Rattus norvegicus genome using SOAPv1.11 software [3]. Clean reads with perfect matches were reserved for further analysis.

The sequences were used to implement a BLASTN search against the miRNA database (miRBase 20.0) to identify conserved miRNAs. The clean reads were compared with the small RNAs annotated in the GenBank and Rfam 10.1 databases to note the small RNA sequences with tag2 annotation software. Before the alignment and annotation, some small RNA tags might map to more than one category. To ensure that every unique short RNA mapped to only one annotation, we follow the following priority rule: rRNAs, tRNAs, snRNAs, snoRNAs, and miRNAs, in which (GenBank>Rfam)>known miRNA>repeat>exon>intron. Some small RNAs not mapped to any annotation information were named as unannotated sequences, and were subsequently used to predict novel miRNAs.

Novel miRNAs were predicted using Mireap software by analyzing the secondary structure, the Dicer cleavage site, and the minimum free energy of the unannotated small RNA tags. Secondary structure prediction was verified using Mfold3.2 software [4], and each sequence was subjected to a further check through the MIREAPv0.2 software [5] according to the following parameter set by Zuker and Jacobson [6]: i) minimal and maximal miRNA sequence length (18–26 nt); ii) minimal and maximal miRNA reference sequence length (20–24 nt); iii) minimal depth of Drosha/Dicer cutting site (3); iv) maximal copy number of miRNAs in the reference (20); v) maximal free energy allowed for a miRNA precursor (−18 kcal/mol); vi) maximal space between miRNA and miRNA* (35); vii) minimal base pairs of miRNA and miRNA* (14); viii) maximal bulge of miRNA and miRNA* (4); ix) maximal asymmetry of the miRNA/miRNA* duplex (5); and x) length of the sequence flanking the miRNA precursor (10). The chosen sequences were transferred into a secondary structure program using RNAfold software. Then, pseudo pre-miRNAs were filtered out by MiPred in accordance with the following rules: minimum free energy >−20 kcal/mol or *P*-value >0.05 [7].

#### 2.2.4 GO annotation and KEGG pathway analysis of the target genes

The GO database was used to enrich for gene functions. Bonferroni correction was used to obtain a corrected *P*-value. GO terms with corrected *P*-value ≤0.05 were defined as significantly enriched in target gene candidates. KEGG is the major public pathway related database [8], and was used to annotate the pathways for target genes and to identify their biological functions. Genes with false discovery rates ≤0.05 were considered as significantly enriched target gene candidates.

#### 2.2.5 Identification of differentially expressed known miRNAs

To identify the differentially expressed miRNAs between the both groups, the 6 small RNA libraries were divided into two groups for statistical analysis after confirming the inter-group correlation. The miRNA expression in the two groups was shown by plotting a Log2-ratio figure and scatter plot. The procedures were as follows: i) normalize the expression of miRNA in the two groups to obtain the expression of transcripts per million (TPM) using the normalization formula: normalized expression=actual miRNA count/total count of clean reads *1×10^6^; ii) calculate the fold-change and *P*-value from the normalized expression. The log2 ratio plot and scatter plot were then generated. The fold-change formula was: fold change=log2 (NAFLD group/normal group). Permut Matrix software with the Pearson distance was used to perform hierarchical clustering of miRNA expression [9].

#### 2.2.6 qRT-PCR validation and data analysis

qRT-PCR was performed to validate the expression of the miRNAs found in the two groups by sequencing. The primers for miRNA amplification were designed on the basis of the miRNA gene sequences in miRBase. Briefly, the reverse transcription reaction was performed according to the SYBR® PrimeScript™ miRNA RT-PCR Kit protocol. All reactions were run in triplicate. A melting curve was constructed using ABI7500 Pro software. Standard curves were established for each assay with serial 5-fold sample dilutions. The PCR efficiencies were calculated based on the slopes of standard curves and the relative amount of each miRNA was determined according to the 2^-ΔΔCt^ method [10]. U6 was used as the housekeeping gene for normalization between samples.

#### 2.2.7 Statistical analysis

The SPSS 17.0 software package was used to process the data and all data were shown with the means±the standard error. Significant differences were examined using one-way ANOVA and the Student׳s t test, and differences with *P*<0.05 were considered statistically significant for all experiments.

## Footnotes

Appendix A Supplementary data associated with this article can be found in the online version at doi:10.1016/j.dib.2016.05.041.
